# mSphere of Influence: Experimental Evolution of RNA Viruses

**DOI:** 10.1128/mSphere.00179-20

**Published:** 2020-03-11

**Authors:** Gonzalo Moratorio

**Affiliations:** a Institut Pasteur de Montevideo, Laboratorio de Evolución Experimental de Virus, Montevideo, Uruguay; b Facultad de Ciencias, Universidad de la República, Laboratorio de Virología Molecular, Montevideo, Uruguay

**Keywords:** virus attenuation, synthetic biology, codon pair bias, mutational robustness

## Abstract

Gonzalo Moratorio works in the field of experimental evolution of viruses. In this mSphere of Influence article, he reflects on how the papers “Virus attenuation by genome-scale changes in codon pair bias” by Coleman et al. (Science 320:1784–1787, 2008, https://doi.org/10.1126/science.1155761) and “Codon usage determines the mutational robustness, evolutionary capacity, and virulence of an RNA virus” by Lauring et al. (Cell Host Microbe 12:623–632, 2012, https://doi.org/10.1016/j.chom.2012.10.008) made an impact on his thinking about how to employ synthetic biology to study experimental evolution of viruses.

## COMMENTARY

About a decade ago, work by Coleman and colleagues (from the Wimmer lab) entitled “Virus attenuation by genome-scale changes in codon pair bias” (1) proposed a strategy to attenuate viruses. This was achieved through the *de novo* synthesis of large DNA fragments, which were synonymously recoded to alter the codon pair bias of viral genes. Thus, the authors designed a variety of constructs or synthetic viruses, without changing the amino acid identity, to examine the question of “to what extent the natural encoding is optimal.” Consequently, they tested these synthetic viruses to suggest this as a strategy to generate live attenuated vaccines. In November 2012, I landed in France to start a postdoc in the Department of Virology at the Institute Pasteur. Only 2 weeks after my arrival and as I was struggling with French, a paper came out using the same viral constructs published by Coleman et al. (1). Even more surprising to me was that I knew the first author (Adam Lauring) with whom I had scientific discussions during an internship I did as a Ph.D. student at the University of California, San Francisco. This was not trivial for someone from my homeland, Uruguay, in a remote corner of the world. Today, I like to think about this coincidence as a sort of signal. In “Codon usage determines the mutational robustness, evolutionary capacity, and virulence of an RNA virus” (2), Lauring and colleagues showed how the synonymous mutations inserted on the aforementioned synthetic viruses impacted the variant distribution of a viral population and define different evolutionary trajectories. Furthermore, they suggested that genetic robustness could be an adaptive trait and that it may play a role in the capacity of an RNA virus to explore different mutational neighborhoods. Together, both papers have significantly impacted my way of thinking about how to employ synthetic biology to address evolutionary hypotheses and design antiviral strategies.

Coleman and colleagues (1) developed a computer algorithm to recode viral genes by modifying the codon pair bias. However, at that time, only a few groups managed to chemically synthesize large DNA molecules without a natural template, opening the door to rewiring living systems. To achieve viral attenuation, the authors increased the frequency of codon pairs of poliovirus that were statistically underrepresented in the host. This approach resulted in synthetic viral genomes with identical antigenic properties (same amino acid sequence) but with a different nucleotide sequence. In a similar fashion, they generated a mutant virus bearing an overrepresentation of codon pairs frequently used by the host. In this way, the authors compared this repertoire of synthetic viruses with the wild-type virus. Both in tissue culture and in the mouse model, the synthetic virus bearing an overrepresentation of “rare” codons (not frequently used by the host) proved to be attenuated. Importantly, by using a luciferase reporter assay, they found a reduced translation of viral proteins. Indeed, this observation was suggested as the main mechanism for the attenuated phenotype. Nevertheless, the attenuated viruses triggered a strong immune response, proving to be sufficient to protect against lethal challenge. A few years later, Lauring and coworkers thought that some of those viral constructs could be used to explore whether codon choice weighs in on defining viral population diversity. In addition, they studied the relationship between the capacity to buffer mutations (mutational robustness) and virulence. To this end, the authors worked with viruses that were designed without altering viral translation, GC content, and RNA folding. One of these synthetic viruses was codon optimized, and the other was designed by codon shuffling the wild-type sequence. They first analyzed the fitness (capacity to produce viable progeny in a given environment) of these variants, then looked at the population diversity by deep sequencing, and finally tested them *in vivo*. They concluded that the codon-shuffled variant was placed in a region of sequence space where mutational neighborhoods were less neutral. Thus, this variant displayed a wider fitness distribution shown to be less robust to mutations. Similar results were observed in the mouse model, where this same variant was shown to be attenuated compared with its counterpart.

Both papers were tremendously inspiring, leading me closer to better understanding how genotype (information) plays on dictating phenotype (function), which determines its evolutionary success (fitness). Indeed, the technology and rationale behind these studies led me to develop my own strategy based on impairing the evolutionary potential of viruses to design antiviral methods (3, 4). Furthermore, these papers were key to my way of thinking about the design of short-term evolution approaches, mostly based on experimentation, to test evolutionary hypotheses. A vast theoretical groundwork is in need of empirical data for validation. In return, biology needs more tangible, mathematical applications of theory to resolve questions that cannot be answered without merging theoretical and empirical research from an interdisciplinary stand point.
